# Bio-processing of macroalgae *Palmaria palmata*: metabolite fractionation from pressed fresh material and ensiling considerations for long-term storage

**DOI:** 10.1007/s10811-020-02295-x

**Published:** 2020-10-19

**Authors:** J. A. Gallagher, J. M. M. Adams, L. B. Turner, M. E. Kirby, T. A. Toop, M. W. Mirza, M. K. Theodorou

**Affiliations:** 1 Institute of Biological, Environmental and Rural Sciences, Aberystwyth University, Gogerddan, Aberystwyth, Ceredigion SY23 3EE UK; 2 Agricultural Centre for Sustainable Energy Systems, Department of Agriculture and the Environment, Harper Adams University, Newport, Shropshire, TF10 8NB UK

**Keywords:** Biorefining, Dulse, Effluent, Rhodophyta, Screw-pressing, Seaweed, Silage

## Abstract

Red algae, belonging to the phylum Rhodophyta, contain an abundance of useful chemicals including bioactive molecules and present opportunities for the production of different products through biorefinery cascades. The rhodophyte *Palmaria palmata*, commonly termed dulse or dillisk, grows predominantly on the northern coasts of the Atlantic and Pacific Oceans and is a well-known snack food. Due to its abundance, availability and cultivation capacity, *P. palmata* was selected for study as a potential candidate for a biorefinery process. In addition to studying juice and solid fractions of freshly harvested *P. palmata*, we have investigated the novel possibility of preserving algal biomass by ensilaging protocols similar to those employed for terrestrial forage crops. In the metabolite partitioning within the solid and liquid fractions following screw-pressing, the majority of the metabolites screened for—water soluble carbohydrates, proteins and amino acids, lipids, pigments, phenolics and antioxidant activity—remained in the solid fraction, though at differing proportions depending on the metabolite, from 70.8% soluble amino acids to 98.2% chlorophyll *a* and 98.1% total carotenoids. For the ensiling study, screw-pressed *P. palmata*, with comparative wilted and chopped, and chopped only samples, were ensiled at scale with and without Safesil silage additive. All samples were successfully ensiled after 90 days, with screw-pressing giving lower or equal pH before and after ensiling compared with the other preparations. Of particular note was the effluent volumes generated during ensiling: 26–49% of the fresh weight, containing 16–34% of the silage dry matter. This may be of advantage depending on the final use of the biomass.

## Introduction

Green biorefining aims to optimize the use of biomass to ensure that resources are fully exploited through the production of various marketable products in coordinated process streams (Yuan and Macquarrie 2015). The development of new fractionation processes in green biorefineries to produce, for example, proteins, bio-based platform chemicals (building blocks made from renewable materials which can be converted to a wide range of chemicals or materials), food additives, therapeutics, fermentation media, animal feed and biofuels from various biomass sources is of increasing importance in the light of concerns over global climate change as the supply of fossil raw materials decreases (Martel et al. 2011; Kamm et al. 2016). Interest is growing in the use of marine algae as feedstocks, as they have high productivity and do not compete with food production for land use and fresh water (Loureiro et al. 2015; Suutari et al. 2015) or require fertilizers (Adams et al. 2017). However, their high water content has energy cost implications that may be inhibitory during biomass preparation processes when working at scale (Suutari et al. 2015; Soomro et al. 2016), as does macroalgae’s rapid decomposition once harvested with ensiling one route to longer-term preservation (Gallagher et al. 2018). Therefore, it is appropriate to investigate both rapid processing and longer-term products from preserved macroalgae. In this study both by ensiling and applying a cost-efficient technique for dewatering used on many land crops, screw-pressing (Takara and Khanal 2011; Kamm et al. 2016).

Large brown macroalgae, especially kelps, are high biomass producing macroalgae commonly considered for biofuel and biorefinery applications. However, these species cannot be significantly dewatered by screw-pressing when fresh (Adams et al. 2017; Gallagher et al. 2017) due to the alginate proportion present. In contrast, *Palmaria palmata* (L.) Weber and Mohr will press successfully upon harvesting to produce a deep red juice (Gallagher et al. 2018). *Palmaria palmata* is a red alga (Rhodophyta) which grows on the northern coasts of the Atlantic and Pacific Oceans and is one of the more popular seaweeds for human consumption. This particularly occurs in the west along the north Atlantic shores of Europe and America (Mouritsen et al. 2013) where it has common names including dulse or dillisk. It has been widely studied over the last 100 years or so (Morgan et al. 1980) and has been shown to contain numerous metabolites of commercial value. It has also been nutritionally considered to be one of the best algal alternatives to cereals in food and feed (Maehre et al. 2014). Techniques for the commercial cultivation of this species have been demonstrated and include growth on longlines (Harnedy et al. 2014) and in land-based tanks, producing relatively uniform algal biomass in a controlled and sustainable manner (Banskota et al. 2014). Although it is less economically viable than kelp species to farm, the *P. palmata* yield reported by Werner and Dring (2011) was 750 g per linear metre of culture string after about 5 months.

*Palmaria palmata* contains a number of carbohydrates identified as having therapeutic qualities, e.g. anti-tumour, anti-viral and anti-coagulant properties (Courtois et al. 2008; Shi et al. 2017), and/or with a commercial value in food processing and industry such as stabilizers, thickeners, emulsifiers and gelling agents (Holdt and Kraan 2011; Mutripah et al. 2014)*.* The algae has a relatively high content of quality protein (21.9% ± 3.5%) in the winter-spring (Galland-Irmouli et al. 1999; Misurcova et al. 2014) and essential amino acids (36.4 g 16 g^−1^ N in one study, 32.1 g 16 g^−1^ N of EAAs in another); (Galland-Irmouli et al. 1999; Misurcova et al. 2014) and the peptides produced by hydrolysis of water- and alkali-soluble protein extracts have also been shown to have therapeutic values (Harnedy and FitzGerald 2013; Harnedy et al. 2014). This species is also noted for its high lipid content with high polyunsaturated fatty acids (49.8% FAME content) and high ω-3 eicosapentaenoic acid content (0.44–0.58% dry weight) in comparison with other macroalgae (van Ginneken et al. 2011; Schmid et al. 2014). The phenolic content of red algae is generally low (< 0.4% dry weight) (Holdt and Kraan 2011), but *P. palmata* has been shown to contain several hydrophilic antioxidant metabolites with potential uses as food additives to improve shelf life (Wang et al. 2010; Farvin and Jacobsen 2013). *Palmaria palmata* also contains photosynthetic pigments which are in increasing demand as natural colourants. Additionally, phycoerythrin, a major red pigment in *P. palmata*, is also reported to have anti-tumour, anti-oxidant, anti-diabetic, immunosuppressive and anti-hypertensive activities (Sekar and Chandramohan 2008; Holdt and Kraan 2011; Dumay et al. 2013). Low-cost screw-pressing, unlike many other methods commonly employed in the laboratory for the extraction of metabolites, can easily be scaled up to industrial level processing (Dumay et al. 2013; Harnedy and FitzGerald 2013). The juice derived from screw-pressing is therefore a possible source of a range of interesting compounds, though the partitioning of metabolites between the press residue and juice will be critical. The other advantage of screw-pressing is that it produces a residue with increased dry matter (DM) content indicating a potential for improved ensiling (Gallagher et al. 2018).

Ensiling provides a longer-term method of preserving the algae biomass and can provide material suitable for biofuel production, such as fermentation to ethanol or anaerobic digestion to methane. Ensiling also causes dewatering through effluent leaching (Johnson et al. 2005) which can remove more water from the biomass prior to processing. Water can also be lost through wilting, and ensiling can be improved through the inclusion of additives.

The study presented here reflects two scenarios, conducted on the same collection of biomass, showing alternative routes for the processing and storage of *P. palmata*. *Palmaria palmata* was screw-pressed when fresh, with the work described here focussing on the partitioning of valuable metabolites and compounds of interest between the liquid and solid fractions generated following screw-pressing. The second explores alternative pre-treatment processing; ensiling additive effects on the silage quality and its dewatering; and the organic acid production of ensiled *P. palmata*. These are important as ensiling does provide longer-term stability of the macroalgae and the dewatering of the biomass may be of an advantage depending on which final use the biomass is planned for. Both scenarios are novel and will contribute to a greater understanding of processing both *P. palmata* and other macroalgae in the future. This in turn will be necessary for planning and establishing biorefining protocols in future using this valuable resource.

## Materials and methods

### Macroalgal material

*Palmaria palmata* was concurrently harvested at low tide from wild stock at Langland Bay, Gower, Swansea, Wales, SA3 4SQ (ordnance survey reference SS 606872), and Bracelet Bay, Gower, Swansea, Wales, SA3 4JT (ordnance survey reference SS 626868), around noon on 16 June 2015. Approximately 16 kg was collected from each bay in a sustainable manner and the 32 kg processed within 2 h of collection. Nine kg was put to wilt (partial drying in air) on a plastic sheet in a ventilated shed. The remaining 23 kg was covered in seawater and stored in a cold room at 4 °C overnight before screw-pressing or chopping on 17 June 2015.

### Screw-pressing

Sixteen kilogrammes of non-wilted biomass was passed through a CP-4 screw-press (Vincent Corporation, Tampa, FL, USA) producing just under a 10-kg solid residue and a liquid termed ‘juice’ which contained small particulates. The juice was sieved to remove large particulates to give a final volume of 3.5 L. This liquid was frozen and stored at − 20 °C prior to analysis. Samples of the solid residue were freeze-dried and stored in a desiccator until metabolite analyses were undertaken.

### Screw-pressed metabolite analyses

Before analyses, the freeze-dried solid residue was milled with an A11 Basic IKA mill (IKA, Germany) to pass through a 1.18-mm sieve. Juice was thawed and centrifuged at 2400*×g*, room temperature for 10 min to remove residual solid fibres. These fibres produced pellets which were combined, washed twice with water and freeze-dried; they are subsequently referred to as ‘juice-pellet’. Mean metabolite concentrations were determined from triplicate extractions. Concentrations for the milled residue were given on a fresh weight (FW) basis using its mean %DM as this gives a more direct comparison with concentrations in the juice.

### Carbohydrates

Water-soluble carbohydrates (WSC) were extracted from the solid residue and the juice-pellet with deionized cold water at room temperature and (separately) with deionized hot water in a boiling water bath for 5 min. The juice was analysed directly. The WSC were quantified using the anthrone colorimetric assay (Yemm and Willis 1954) at a titre-plate scale (Farrar et al. 2012) against galactose rather than fructose standards.

### Proteins and free amino acids

Proteins were extracted from the solid residue and the juice-pellet with deionized water, 0.1 M sodium hydroxide or 1% sodium dodecyl sulphate (SDS) in a shaking incubator at 25 °C for 3 h and then quantified against bovine serum albumin standards through the Lowry method (Lowry et al. 1951) at a titre-plate scale. The juice was analysed directly. Free amino acids were extracted from the solid residue (average 15.8 mg: 1 mL) and the juice-pellet (average 7.4 mg: 1 mL) by steeping in 100% methanol overnight at 4 °C in the dark. The juice was also diluted (1 mL: 3.5 mL) with 100% methanol and stored overnight at 4 °C in the dark. Total free amino acids were measured using the ninhydrin assay (Yemm and Cocking 1955) as modified by Ferguson and Sims (1974), but also replacing 2-methoxyethanol with ethanol. Glutamate and leucine were used as standards and produced equal reactions; a mean standard curve was calculated for quantification.

### Lipids

Lipid analysis was by gravimetric means and was based on the general method of Folch et al. (1957) for total lipid extraction, though modified to minimize the co-extraction of chlorophyll (Zhao et al. 2018). The solid residue was extracted twice with acidified 2:1 dichloromethane:methanol (sulphuric acid was added to the solvent to a final concentration of 0.1 M H_2_SO_4_). Solid material was removed by centrifugation and the supernatant phase-separated with 0.9% sodium chloride. The upper aqueous phase was discarded and the interface was washed three times with 1 mL 1:1 methanol:water. The lower phase was transferred to a glass evaporating dish and dried. The juice was acidified to a final concentration of 0.1 M sulphuric acid and then mixed with methanol and dichloromethane in the ratio of 3:12:5 to give a homogeneous solution. This was phase-separated by the addition of dichloromethane (5 parts) and deionized water (5 parts) and then processed as above, except that any coagulated material on the interface was carefully removed before washing the interface with methanol:deionized water. The dried lipid samples were further extracted into two 5-mL washes of hexane, which were transferred to pre-weighed glass dishes and dried to constant weight. The final lipid samples had a negligible observable green colour.

### Pigments

Pigments were analysed in the solid residue and the juice-pellet by steeping overnight at 4 °C in the dark with the extraction mediums (detailed below). The juice was also diluted with the extraction mediums and left overnight at 4 °C in the dark.

Phycoerythrin was extracted in 0.1-M potassium phosphate buffer, pH 6.5. Spectra between 350 and 750 nm and absorbance at 564, 618 and 730 nm were recorded with a UV/Vis spectrometer UV4 (UNICAM, now Thermo Fisher Scientific, USA) running VisionPro software, which was used to also calculate derivatives of the spectral curves to confirm phycoerythrin presence before quantifying using the equation within Sampath-Wiley and Neefus (2007).

Chlorophylls and carotenoids were extracted in 100% methanol and absorbance recorded at 470 nm, 653 nm and 666 nm. Quantification was produced from these absorbances using published equations (Lichtenthaler and Wellburn 1983).

### Phenolics and antioxidants

The solid residue and the juice-pellet were extracted in duplicate biological replicates by steeping in deionized water overnight at room temperature in the dark. The extracts were combined and stored at − 20 °C. The juice was diluted as necessary and analysed directly. Phenolic acids were quantified by the Folin-Ciocalteu reaction against a gallic acid standard and expressed as mg gallic acid equivalents. Antioxidant activity was assessed by diphenylpicrylhydrazyl (DPPH) scavenging capacity (Farvin and Jacobsen 2013) measured at 517 nm on the same extracts. Sample dilution curves including minus sample controls (for zero scavenging) and paired minus reagent blanks (for background absorbance) were analysed, and scavenging capacity (%) is calculated as shown in Eq. 1:

1 $$ Capacity\ \left(\%\right)=\left(1-\left(\frac{\mathrm{sample}\ {A}_{517}\hbox{--} \mathrm{reagent}\ \mathrm{blank}\ {A}_{517}\Big)\ }{\mathrm{sample}\ \mathrm{control}\ {A}_{517}\hbox{--} \mathrm{reagent}\ \mathrm{blank}\ {A}_{517}}\right)\right)\times 100 $$

The data are expressed as EC_50_ values (the sample concentration required to give 50% DPPH scavenging). The EC_50_ for the reference compound ascorbic acid was 5.7 μg mL^−1^ under the same conditions.

### Ensiling pre-treatments

Approximately 7 kg of *P. palmata* stored in seawater overnight and the 9 kg (initial weight) of wilted *P. palmata* were chopped with a garden shredder (Viking GE-250 Stihl, UK) to discrete pieces that were approximately 3 × 2 cm mimicking the particle size of screw-press residue material. These, and the solid residue minus samples from the 16 kg (initial weight) screw-pressed *P. palmata*, were then used to produce ensiled material for the storage study.

### Ensiling

Safesil (Kelvin Cave Ltd., Langport, UK) is a terrestrial crop silage additive manufactured to contain a mixture of the food-safe preservatives, sodium benzoate, sodium nitrate and potassium sorbate. Biomass from each pre-processing technique were labelled as SP, screw-pressed; Ch, chopped only; WCh, wilted, then chopped. Each pre-processing sample was divided into two and treated with either Safesil silage additive (A) or an equivalent amount of tap water (control, C) prior to ensiling. Therefore, the six different treatments were SP-A, SP-C, Ch-A, Ch-C, WCh-A and WCh-C. Both the additive and control treatments were applied at the recommended dosage rate of 4 mL kg^−1^ FW for the Safesil product.

To produce the silage, 3-kg batches of each pre-processing sample were spread onto plastic sheeting and sprayed uniformly with Safesil (A) or water (C) applications, shaken within the sheet for 2 min and resprayed with three mixing and spraying replications in total.

Immediately after the spray treatments, triplicate 500 g samples of each treated biomass were sealed in 160-μm-thick embossed food grade vacuum seal bags (30 × 50 cm; Lava, Germany) using a vacuum packaging laboratory-scale ensilage process similar to that described by Johnson et al. (2005). A V400OP commercial vacuum packer (Lava) was used to remove air from the bags to a pressure of − 0.9 bar and triple heat sealed before being stored at approximately 15 °C for 90 days. Sub-samples from each of the treatments were removed after the addition of Safesil or water but prior to ensiling, with fractions analysed immediately or stored at − 20 °C prior to subsequent analyses.

### Silage analyses

**Fig. 1:**
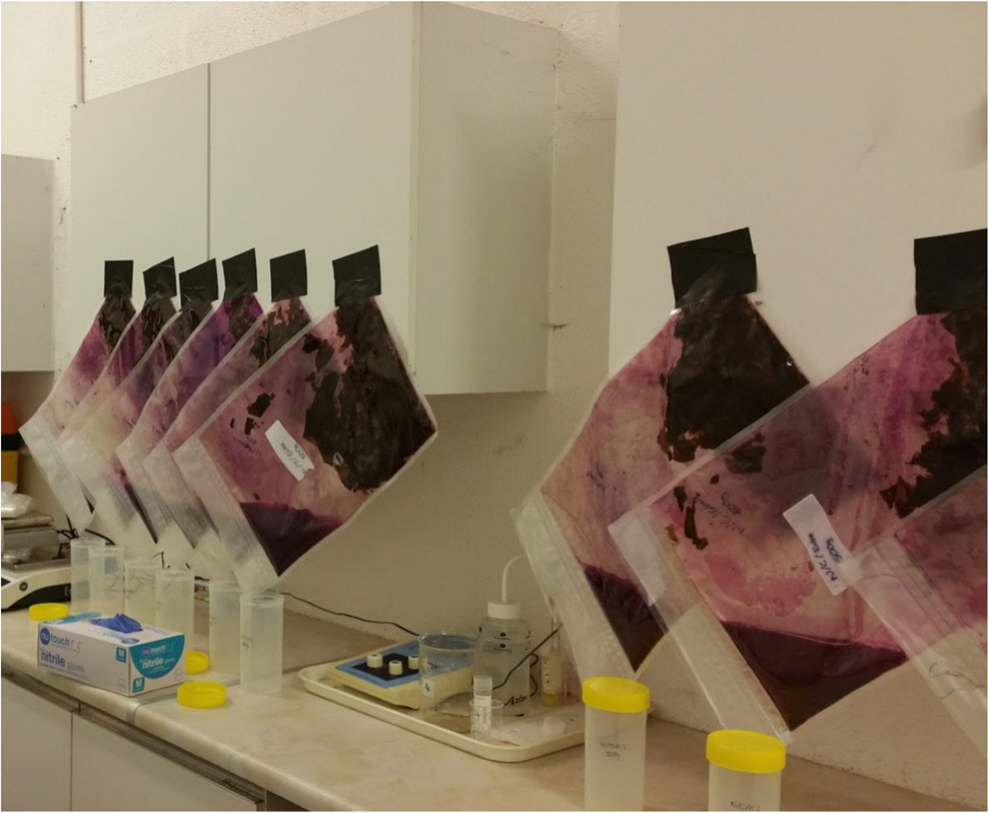
Day 90 ensiled seaweed demonstrating the volume of liquid effluent produced per treatment

On day 90 of ensilage, bags were inverted to drain the large volume of effluent from the solids (Fig. 1), with the lowest corners cut and the collected effluent volumes recorded. The DM content of the solid and effluent fractions were determined by oven drying 4 g samples (duplicate samples per bag) to constant weight at 60 °C using a UF260 oven (Memmert, Germany).

### Ensiled solid and effluent pH measurements

The pH of the ensiled solid fraction was determined for each replicate silage bag. A 10-g sample was weighed into a Stomacher bag (Seward, Worthing, UK) with 90 g of deionized water. The sample was pummelled for 3 min at 230 rpm using a Stomacher 400 Circulator (Seward) and the pH measured immediately using a calibrated Jenway 3505 pH meter with temperature probe (Cole-Palmer, UK). The previously separated silage effluent fraction was measured directly using the same pH and temperature probe.

### Lactic acid and other volatile acids

Compositional analyses of the ensiled solid fraction were determined by weighing a 20 g sample into a new Stomacher bag with 100 g of high-performance liquid chromatography grade water. The sample was pummelled as detailed above and the liquid removed for analysis. The liquid obtained from solid extracts and the liquid effluent fraction from the ensilage bag were filtered through a 25-mm, 0.45-μm nylon syringe filter (Chromatography Direct Ltd., UK) into Low EVap Filter Vials (Thomson Instrument Company, USA) with 3-methylpentanoic acid used as an internal standard at a final concentration of 1 mg mL^−1^. The vial was capped and vortexed for 30 s. Analysis was performed using an Agilent 1100 HPLC system (Agilent Technologies, UK) consisting of binary pump, solvent degasser, autosampler, column oven and diode array detector. Method conditions consisted of a 0.6 mL min^−1^ flow rate of 5 mM sulphuric acid through an Aminex HPX-87H column (BioRad Laboratories Ltd., UK) maintained at 55 °C with UV detection at 210 nm. Internal standards were used to detect succinic, lactic, formic, acetic, propionic, isobutyric, butyric, isovaleric and valeric acids in liquid samples. Individual samples from solid and effluent fractions were analysed per triplicate set of bags, each analysed twice from the same HPLC vial.

### Statistical analyses

Data were analysed using GenStat edition 18 (VSN International, UK). Metabolite concentrations were analysed by one-way analysis of variance. Two-way analysis of variance was used to determine significances for silage composition analyses (pre-processing techniques × additive type). Differences between means were assessed with Fisher’s protected least significant difference (*P* ≤ 0.050).

## Results

### Metabolite fractionation of *P. palmata* by screw-pressing

The concentrations of the major classes of algal metabolites were measured in the solid residue, clarified juice and juice-pellet obtained from the screw-press using predominantly spectrophotometric assays (Table 1). Because the yields of the fractions differed between categories, the percentage of the total biomass content recovered in these fractions is also of interest and is therefore included in Table 1. The ash content for each fraction was determined as 66.1% ± 0.64 std. dev. dry solids with no significant differences seen between any treatment (*n* = 6, *P* < 0.001, analysis not shown).

**Table 1:** Metabolite content of screw-press residue, juice fractions and total yield partitioning between the fractions

Metabolite	Material					Yield	
	Residue per g dry weight	Residue per g fresh weight	Juice per mL	Juice-pellet per g dry weight	*P* value	Residue (% total)	Juice + pellet (% total)
Carbohydrates—WSC							
Cold water soluble (mg)	85.91	15.32^c^	10.30*^b^	0 ^a^	< 0.001	80.7	19.3
Hot water soluble (mg)	99.75	17.78^b^	10.30*^a^	5.74 ^a^	0.012	82.9	17.1
Proteins/amino acids							
Water-soluble protein (mg)	24.87	4.43^b^	1.04*^a^	0 ^a^	< 0.001	92.4	7.6
Alkali-soluble protein (mg)	44.07	7.85^a^	1.04*^a^	94.38 ^b^	< 0.001	94.8	5.2
SDS-soluble protein (mg)	41.26	7.34^a^	1.04*^a^	16.24 ^b^	0.018	95.1	4.9
Soluble amino acids (μmol)	44.73	7.96^a^	9.24^b^	20.26 ^c^	< .001	70.8	29.2
Lipids							
Total (mg)	3.28	0.58	0.05	n.d.	0.012	97.1	2.9
Pigments							
Phycoerythrin (mg)	6.078	1.082^b^	0.071^a^	0.110^a^	< 0.001	97.7	2.3
Chlorophyll *a* (mg)	1.502	0.267^b^	0.010^a^	1.801^c^	< 0.001	98.2	1.8
Total carotenoids (mg)	0.300	0.053^b^	0.002^a^	0.757^c^	< 0.001	98.1	1.9
Phycoerythrin: chlorophyll *a*	4.05	4.05^b^	6.99^c^	0.06^a^	< 0.001	─	─
Phenolics							
Total (mg gallic acid equivalents)	2.79	0.50^b^	0.16^a^	1.31^c^	< 0.001	89.5	10.5
Antioxidant activity							
DPPH radical scavenging: EC_50_	9.4 mg mL^−1^	52.6 mg mL^−1^	213.1 μL mL^−1^	AQL	< 0.001	92.0	8.0

Measured concentrations for milled residue on a dry weight are presented for information but were not included in ANOVA. Only means for direct carbohydrate and protein measurements on juice are presented. These may be most like the water extraction methods but are included and compared with the different extractions of solid residues. Means in rows followed by the same superscript letter are not significantly different at the 5% level according to Fisher’s protected least significant difference. Relative recovery in the two fractions as %total material recovered from the screw-press is also shown. *n.d.* not determined, *AQL* above quantifiable level, *replicate values within category due to the liquid nature of the product, allowing comparison with other fractions. *n* = 3 throughout

Most of the carbohydrate detected by the anthrone assay and measured as galactose equivalents was released from the solid residues with cold water. Using hot water to extract released a small proportion of additional WSC, increasing the measured WSC content from 15.32 mg g_FW_^−1^ (cold extract) to 17.78 mg g_FW_^−1^ (hot extract). In contrast, the juice-pellet released no WSC in a cold extract, but did contain 5.74 mg g_DW_^−1^ after a hot extraction. The juice was measured directly, and its production was most similar to the cold process, but the quantity released (10.3 mg mL^−1^) has been replicated in Table 1 in the hot extraction row too to allow comparisons to be made with the other fractions. The carbohydrate content of the juice was approximately two-thirds that of the solid residue, equating to a recovery of just under 20% of the total WSC.

Protein was found in all fractions (Table 1). Just over half the protein in the solid residue was water soluble; alkali and the detergent SDS were almost equally effective at releasing the remainder. The juice-pellet did not contain water-soluble protein but did contain significant amounts of non-water-soluble protein, particularly protein amenable to release with alkali. Due to the liquid nature of the juice fraction, only one direct measurement of protein concentration is possible for juice. This number has been replicated in Table 1 under the different soluble protein categories as above to allow comparisons with the other materials analysed. The juice contained 1.04 mg mL^−1^ protein, approximately one quarter of the water-soluble concentration found in the solid residue on a FW basis. This equated to recovery of 7.6% of total biomass water-soluble protein in the juice. The concentration of free amino acids was slightly, but not significantly, higher in the juice than in the solid residue on a FW basis. This resulted in a much higher fractionation, with nearly 30% partitioning into the juice (Table 1). Preliminary investigation of the amino acid complement of the juice by HPLC-MS (with identity based purely on nominal mass and ion fragmentation spectra) showed the presence of the essential amino acids lysine, histidine, phenylalanine and leucine plus also glutamate, aspartate and proline; collectively, these are presented as ‘soluble amino acids’ in Table 1.

Lipids were not measured in the juice-pellet as insufficient material was available for the scale of gravimetric analyses but were found in both the solid and juice fractions. The solid residue contained 0.58 mg g_FW_^−1^ (Table 1). Significantly less lipid (*P* = 0.12) was present in the juice, only 0.05 mg mL^−1^ which equated to recovery of 2.9% total biomass lipid from pressing.

Compounds from three classes of pigment (phycobiliprotein, chlorophyll and carotenoid) from the light harvesting complexes found in red algae were measured. The pigment with the highest concentration in juice was the phycobiliprotein phycoerythrin (Table 1). The juice contained very little chlorophyll or carotenoid, though significant concentrations of these were present in the juice-pellet. Nevertheless, overall less than 3% of the pigments present in the biomass was released into the juice by screw-pressing.

There were highly significant (*P* < 0.001) differences between the concentrations of phenolic compounds between the three fractions (Table 1). The juice had the lowest concentration of phenolics, with just over 10% partitioned into the juice by screw-pressing. Antioxidant activity was assessed by DPPH scavenging capacity, and EC_50_ values are presented (Table 1). The fraction/extract concentration required for EC_50_ was higher for the juice than for the solid residue; there was more activity in 1 g residue than in 1 mL juice. Interestingly, no detectable decolouration was measured in the juice-pellet fraction, meaning that the EC_50_ is listed as above quantifiable limit (AQL) in Table 1. The partitioning of total biomass antioxidant activity by screw-pressing was calculated based on the total volume of EC_50_-strength solution that could be prepared. The proportion of antioxidant activity in the juice fraction was lower than for phenolic content.

### Silage quality and silage effluent production

The initial pH of the screw-pressed, chopped and wilted-chopped (SP, Ch and WCh) algal biomass, immediately after their treatment with Safesil silage additive or water as control, ranged from pH 6.4 to 7.0 (Table 2). At day 0, there were no significant two-way interactions on pre-processing methods and additive application; however, there were separate pre-processing and additive effects. Screw-pressing significantly decreased pH, whilst the control silages also had a significantly decreased pH (Table 2). After 90 days of incubation in vacuum pack bag silos in both a visual inspection and from the perspective of pH decline, all macroalgae preparations appeared to ensile well. One point of note was the large volume of effluent present in all bags (Fig. 1). The pH values recorded for the silage solids and corresponding liquid effluent fractions (Table 2) were all below the pH 4.4 level which is considered the highest pH for satisfactory ensiling of land-based herbage within 7 days (Black 1955). Statistical analysis on the pH values revealed significant differences between treatments, with pH values demonstrating similar patterns of pH change in both the solid silage and the generated effluent.

**Table 2:** Silage pH and dry matter distribution within the solid and effluent fractions of differently prepared *P. palmata* silages

	Treatments						Standard error			*P* values		
Analyses	SP-C	SP-A	Ch-C	Ch-A	WCh-C	WCh-A	Pre-process	Additive	Pre-process × additive	Pre-process	Additive	Pre-process × additive
pH												
Pre-ensiling	6.37^Aa^	6.73^Ab^	6.73^Ab^	7.00^Bb^	6.63^Ba^	7.03^Bb^	0.03	0.03	0.05	< 0.001	< 0.001	0.340
Solid (ensiled)	3.58^a^	3.57^a^	3.98^c^	3.61^a^	3.76^b^	3.69^b^	0.02	0.01	0.02	< 0.001	< 0.001	< 0.001
Effluent (ensiled)	3.75^a^	3.74^a^	4.20^c^	3.75^a^	3.99^b^	3.89^b^	0.02	0.02	0.03	< 0.001	< 0.001	< 0.001
Effluent produced												
Effluent produced (mL kg_FW_^−1^)	267^a^	323^b^	490^d^	447^c^	453^cd^	463^cd^	9.53	7.78	13.47	< 0.001	0.493	0.010
DM (g kg_FW_^−1^)												
Solid	209.8^b^	203.5^a^	220.1^b^	203.7^a^	230.5^b^	206.0^a^	5.04	4.12	7.13	0.300	0.019	0.463
Effluent	130.4	130.2	101.9	142.7	122.1	129.8	9.20	7.51	13.02	0.828	0.157	0.286
Effluent proportion of silage by DM (%)	16.7^A^	21.8^A^	29.6^B^	34.3^B^	28.0^B^	29.4^B^	1.99	1.62	2.81	0.002	0.136	0.785

*SP* screw-pressed, *Ch* chopped only, *WCh* wilted then chopped, *A* Safesil additive, *C* water control. *n* = 3. Means in rows followed by the same letter are not significantly different at the 5% level according to Fisher’s protected least significant difference. Where no two-way interaction is present, significances relating to pre-processing technique are represented by capital superscripts, and significances relating to additive effect are represented by lower case superscripts

**Fig. 2:**
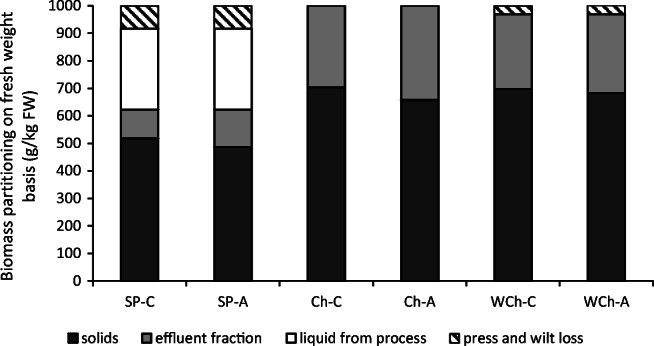
Fresh weight biomass partitioning from *P. palmata* after different pre-processing methods and ensiling. Biomass partitions of the fresh weight (FW) of *P. palmata.* Losses of fresh weight was due to screw-pressing and wilting losses. White, juice from screw-pressing; light grey, silage effluent; dark grey, solid fraction of the silage; cross-hatched, losses through screw-press and wilting; SP, screw-pressing pre-processing; Ch, chopped only; WCh, wilted, then chopped; −C, water only added in silage preparation; −A, Safesil silage additive added in silage preparation

The relative proportions of *P. palmata* following the screw-pressing, wilting and chopping pre-treatments can be seen in Fig. 2. Screw-pressing lost 84 g kg_FW_^−1^ of the material in the press, but produced 294 g kg^−1^ of the FW as a juice. Following ensiling, a further 104 g kg^−1^ or 136 g kg^−1^ (for SP-C and SP-A respectively) of the initial fresh weight was lost as effluent, making the ensiled solid fraction 518 g kg^−1^ or 486 g kg^−1^ (for SP-C and SP-A respectively) of the initial FW with the rest of the weight distributed into the effluent, juice or lost in the initial screw-press process. Material that was chopped did not lose any weight through this process and wilting overnight lost a mere 32 g kg_FW_^−1^. Effluents from these pre-processing steps were thus unsurprisingly larger than those from the screw-press with 296 g kg^−1^ (Ch-C), 343 g kg^−1^ (Ch-A), 271 g kg^−1^ (WCh-C) and 285 g kg^−1^ (WCh-A) FWs becoming effluent. The volume of effluent produced from each *P. palmata* sample was considerable compared with that expected from ensiled terrestrial crops using the same ensiling technology (Table 2). Significantly (*P* = 0.010) less effluent was produced from SP-C followed by SP-A, as expected by the process, with larger quantities of effluent produced from treatments Ch-A, WCh-C, WCh-A and Ch-C respectively (Table 2).

Although the solid proportions in each of the ensiled treatments differed, ranging from 657 (Ch-A) to 704 (Ch-C) g kg_FW_^−1^ (Fig. 2), when expressed on a DM basis following the ensiling process, there was no significant difference between the total of the solid and effluent DM values in each treatment bag. Treatment DM contents ranged from 161.0 to 176.3 g kg_FW_^−1^, though there was a significant (*P* = 0.019) effect of the Safesil additive on the DM content of the ensiled solid fraction, with a higher DM content noted for control treatments than for those treated with Safesil (Table 2). Within the total DM contents, approximately one-third of the total DM fractionated into the silage effluent within the 90 day ensiling period. There was a significant (*P* = 0.002) effect of pre-processing technique on DM conversion to effluent, with screw-pressing producing significantly less DM conversion to effluent, compared with both Ch and WCh treatments. There was no significant effect of additive or two-way interactions.

The silages contained large proportions of organic acids (Table 3). Within the solid fraction, the main acids were lactic acid (no significant difference between treatments) and acetic acid which was significantly higher (*P* < 0.001) for Ch-C. There were low detectable concentrations of succinic and formic acids in the effluent fractions, not seen in the solids; for isobutyric and isovaleric acid low concentrations were seen in the solids with higher concentrations in the effluent.

**Table 3:** Organic acid presence in silage fractions of *P. palmata*

Analyses	Treatments						Standard error			P values		
	SP-C	SP-A	Ch-C	Ch-A	WCh-C	WCh-A	Pre-process	Additive	Pre-process × additive	Pre-process	Additive	Pre-process × additive
Lactic acid (g kg_DM_^−1^)												
Solid	30.1	37.9	23.7	26.2	25.6	32.2	2.60	2.12	3.68	0.086	0.085	0.760
Effluent	169.3^Bb^	145.8^Ba^	142.4^Ab^	112.8^Aa^	143.6^Ab^	130.7^Aa^	5.49	4.48	7.76	0.007	0.005	0.569
Total	199.3^B^	183.7^B^	166.1^A^	139.0^A^	169.2^A^	162.9^A^	7.07	5.77	9.99	0.007	0.068	0.595
Acetic acid (g kg_DM_^−1^)												
Solid	9.2^a^	10.3^a^	22.7^c^	5.9^a^	16.1^b^	10.8^ab^	1.22	1.00	1.73	0.051	< 0.001	< 0.001
Effluent	49.6^ab^	39.7^a^	125.1^c^	38.6^a^	67.0^b^	44.2^ab^	5.28	4.31	7.46	< 0.001	< 0.001	< 0.001
Isobutyric acid (g kg_DM_^−1^)												
Solid	13.5	15.0	16.9	10.7	15.5	19.2	1.36	1.11	1.93	0.186	0.837	0.059
Effluent	67.4^bc^	56.7^ab^	87.6^d^	48.4^a^	73.4^c^	67.6^bc^	3.07	2.51	4.34	0.179	< 0.001	0.005
Isovaleric acid (g kg_DM_^−1^)												
Solid	3.6^A^	2.0^A^	5.7^AB^	3.3^AB^	5.7^B^	8.3^B^	1.01	0.82	1.43	0.034	0.694	0.221
Effluent	27.6	21.2	34.7	21.7	29.4	27.1	3.06	2.50	4.33	0.604	0.062	0.478
Succinic acid (g kg_DM_^−1^)												
Effluent	2.3^b^	0.7^ab^	0.0^a^	1.5^ab^	4.2^c^	2.0^b^	0.41	0.33	0.57	0.004	0.116	0.017
Formic acid (g kg_DM_^−1^)												
Effluent	2.83^b^	1.5^a^	4.2^b^	0.9^a^	3.0^b^	1.3^a^	0.42	0.34	0.59	0.737	< 0.001	0.233

*SP* screw-pressed, *Ch* chopped only, *WCh* wilted then chopped, *A* Safesil additive, *C* water control. *n* = 3. Means in rows followed by the same letter are not significantly different at the 5% level according to Fisher’s protected least significant difference. Where no two-way interaction is present, significances relating to pre-processing technique are represented by capital superscripts, and significances relating to additive effect are represented by lower case superscripts

Analyses of the DM content of the effluent fraction showed that there was no significant difference between any of the treatments. However, the same significant (*P* < 0.001) pattern of pH change (Table 2) noted in the solid fraction was reflected in the effluent fraction. A wider range of acids were found in the effluent fraction and the concentrations were considerably greater than in the solid fraction. Lactic acid was the predominate acid, producing significantly (*P* < 0.050) higher concentrations for SP, followed by Ch and WCh, and also for those prepared as controls compared with those with Safesil (A). However, there was no two-way interaction for lactic acid. Acetic acid was the second highest concentration across treatments, followed by isobutyric and isovaleric acids. Treatment Ch-C had significantly (*P* < 0.050) higher concentrations of all three acids, followed by WCh-C, WCh-A and SP-C, and then SP-A and Ch-C. Solid and effluent samples were also screened for propionic, butyric and valeric acids, but these were not detected in either sample type (data not shown).

## Discussion

Red algae belonging to the phylum Rhodophyta have perhaps been under-exploited in a biorefinery context in the past, as their productivity is lower than for some of the larger brown algae (Werner and Dring 2011). However, they do contain an abundance of useful chemicals, including bioactive molecules and present opportunities for the manufacture of different products in biorefinery cascades. Due to its abundance, availability and ease of cultivation, we have chosen to study *P. palmata* as a potential candidate for a biorefinery process. In addition to studying juice and solid fractions of freshly harvested *P. palmata*, we have investigated the novel possibility of preserving algal biomass by ensilaging protocols similar to those employed for terrestrial forage crops.

### Metabolite partitioning within screw-press fractions

The removal of useful metabolites from biomass within a biorefining scenario, prior to bulk processing of residues for lower-value products such as sustainable fuel production, can have considerable economic advantages (Francavilla et al. 2015). In this study, bulk screw-pressing of fresh *P. palmata* biomass was successfully tested at pilot scale, although the juice appeared to be more dilute than when pressed at bench top scale (Gallagher et al. 2018). Whilst it may be possible to increase the volume and/or the concentration of the juice produced during screw-pressing by adjusting the back pressure, surface water present on the feedstock would also have played a part as the material tested at bench top scale was dried more thoroughly before screw-pressing. Recovery of pressed product was higher in the current study at 84% rather than 68% at bench top scale (Gallagher et al. 2018) but partitioning of the utilized products into juice lower at 26% juice and 74% residue rather than 37% and 63%, respectively. The major proportions of the original biomass metabolites measured in this study remained in the screw-pressed residue. This is desirable in a biorefining process as the removal of a significant proportion of the juice close to the harvest site would result in the production of a more concentrated, more transportable material for further processing than the original whole material.

The juice-pellet was high in alkali-soluble protein, chlorophyll and carotenoids. These could have originated from plastid membranes, but only constituted a very small proportion of the recovered biomass. In general, non-water-soluble metabolites partitioned poorly into juice, suggesting that the juice was almost entirely expressed cell sap. The juice contained good concentrations of water-soluble metabolites, being relatively high in WSC, amino acids and phenolic compounds that may have potential value. These areas are discussed in more detail below.

### Carbohydrates

Many marine macroalgae are high in carbohydrate, both as structural and soluble intracellular compounds. These are predominantly polysaccharides, and many are useful as stabilizers, thickeners and emulsifiers, although the nature of the polymers present does vary widely between different types and species of seaweed (Holdt and Kraan 2011; Mutripah et al. 2014). This study concentrated on 5-min extractions with water as the most likely to provide useful comparisons between the WSC content of SP juice and the residue fractions. In general, many of the larger insoluble structural polysaccharides of macroalgae require lengthy extraction procedures with aggressive media or appropriate enzyme preparations to break down algal structures prior to solubilization. In the current study, all the readily soluble carbohydrate was extractable from the solid residue with water, as extraction with sodium carbonate or sodium hydroxide (Jiao et al. 2012) for 5 min did not significantly increase the measured values (data not shown).

The main storage carbohydrate in *P. palmata* is floridoside (Martinez and Rico 2008). This 1-linked galactose (Lahaye and Vigouroux 1992) has been reported to be present in high concentrations in this species (Martinez and Rico 2008). Endogenously floridoside is thought to be the main sugar involved in osmoregulation (Simon-Colin et al. 2002). Therapeutically, it is reported to be a modifier of the immune system with potential anti-tumour activity (Courtois et al. 2008).

Several larger polymers are reported to be abundant in *P. palmata* (Mutripah et al. 2014). Carrageenans are components of algal cell walls made up of galactose and 3,6-anhydrogalactopyranose, but despite being high-molecular-weight polysaccharides, most have been shown to be extractable with short treatments in hot water. They may be a component of the additional carbohydrate proportion removed by hot water over a cold water extraction in this study. They could also be present in the juice if elevated temperatures are experienced in the screw-press. They have widespread applications in the food industry due to their useful gelling properties (Holdt and Kraan 2011). The other main polysaccharide within *P. palmata*, constituting up to 35% dry weight, is xylan. Composed of β-(1-3) and β-(1-4) linked D-xylose units containing no sulphate, ester or methoxyl groups, these structural polysaccharides are known to have some water solubility (Percival and Chanda 1950). Studies have previously shown galactose to be the only or the major sugar component present in carbohydrates extracted from *P. palmata* with water after non-osmotically stressful treatments (Lahaye and Vigouroux 1992), so floridoside (1-linked galactose) may reasonably be assumed to be the major source of the carbohydrate component of the juice produced by screw-pressing.

### Protein and amino acids

*P. palmata* has frequently been noted for its high protein content (Galland-Irmouli et al. 1999; Maehre et al. 2014), perhaps commonly as high as 20% and sometimes up to 35%, which is comparable with more traditional sources of protein like chicken and other meats (Dumay et al. 2013; Mouritsen et al. 2013). However, values of this order have generally only been reported when crude protein has been estimated from measurements of total nitrogen (crude protein = 6.25 × N). Bjarnadottir et al. (2018) consider that this method is likely to considerably over-estimate the actual protein content in *P. palmata*. Certainly, studies which measure protein content directly report lower values (Schiener et al. 2017). This study would support that the protein content of the solid material was just under 5%, comparable with the 11% protein in Schiener et al. (2017) when taking effects of seasonal variation into account. *P. palmata* protein content in the summer was around half that found in the winter (Galland-Irmouli et al. 1999) which is a result of much higher carbohydrate concentrations during the growing season (Adams et al. 2011a; b; Schiener et al. 2015). The protein of *P. palmata* is considered to be a good source of several essential amino acids relative to the recommended daily intake, particularly for lysine (Maehre et al. 2014; Misurcova et al. 2014). Both glutamate and aspartate, which can be used in the food industry to confer seasoning properties, are also major components (Mouritsen et al. 2013). When screw-pressing, the proteins of *P. palmata* biomass did not partition well into the juice fraction, although water-soluble protein was unsurprisingly better than less soluble proteins. However, free amino acids partitioned particularly well with nearly 30% of the total tissue content found in the juice, and preliminary evidence indicated that several essential amino acids (lysine, histidine, phenylalanine and leucine) were present.

### Phenolics

The phenolic content of red algae is generally lower than that of brown algae (Holdt and Kraan 2011), and the concentration of phenolic compounds measured in this study was of the same order as that reported by Schiener et al. (2017). Phenolic compounds often have antioxidant properties, and *P. palmata* has been reported to have a range of classes of hydrophilic antioxidant components (Wang et al. 2010). Consequently, measurements of phenolic content can show correlations with antioxidant activity, but the relationship is variable, and other compounds can be involved (Farvin and Jacobsen 2013; Machu et al. 2015). In this study, the phenolic compounds present in the juice-pellet did not result in any detectable antioxidant activity. The juice had one-third the concentration of phenolics as the solid residue, but the half maximal effective concentration (EC_50_) was four times higher. Therefore, the relative antioxidant activity of the compounds in the juice was lower than of those remaining in the residue. Nevertheless, compounds with useful antioxidant and radiation protection activities may be present in the juice, and natural antioxidants have a considerable value as food additives to improve shelf life (Farvin and Jacobsen 2013).

### Ensiling process

The manufacturers claim that the additive (Safesil) lowers crop pH, inhibits potential spoilage microorganisms, reduces silage effluent production and enhances the aerobic stability of terrestrial crop silages (Kelvincave 2020). The addition of Safesil to Ch *P. palmata* did significantly improve the ensiling process for this pre-processing treatment, relative to the addition of water as a control, producing a lower pH (solid and effluent fractions), lower acetic (solid fraction) and volatile fatty acids (effluent fraction) concentrations. However, when SP and WCh *P. palmata* were treated with Safesil, similar improvements were not detected compared with the water controls. Therefore, we conclude that an addition of Safesil does not benefit the ensiling of *P. palmata* and the pre-processing techniques of screw-pressing or wilting appear sufficient at improving the ensiling process.

All pre-processing methods, with and without the addition of Safesil, produced an ensiled product for longer-term storage of this readily degradable biomass. Overall, there was no difference in total DM content of each silage (solid and liquid fraction combined); however, the solid fraction demonstrated a significantly higher DM for the control compared with Safesil treatments. For all treatments, a large percentage of the DM moved from the biomass into the effluent fraction after 90 days of ensiling. The effluent produced from the screw-pressed silage unsurprisingly produced a lower quantity of effluent compared with the Ch and WCh treatments. The screw-pressing pre-treatment also significantly reduced the pH of the biomass prior to ensiling on day 0. However, by day 90 of ensiling, the pH had reduced to satisfactory silage levels for all, though the screw-pressed samples remained the lowest for the residue and lower or equal to ChA for the effluent. The high effluent production from *P. palmata* could be both beneficial and problematic for future biofuel use. Through effluent production, DM and other components were lost from the solid fraction into the effluent that may affect the energy potential of the solid biomass fraction. For example, if the solid ensiled *P. palmata* was to be used for anaerobic digestion, the methane potential of the ensiled biomass would be lower as some of the utilizable methanogenic substrates (particularly acetic acid) of the biomass would transfer to the effluent. Herrmann et al. (2015) demonstrated that methane yield of the seaweed biomass could only be preserved if the effluent was anaerobically digested along with the ensiled seaweed. Alternatively, the effluent could be seen as a relatively high DM flowable feedstock, suitable for continuous fermentation encompassing membrane filtration systems. Further work is required to identify and quantify the compounds present in both *P. palmata* silage and the effluent and to ascertain their potential as metabolite sources.

## Conclusions

Red algae have perhaps been under-utilized in the past, as their productivity is lower than for some of the larger brown algae (Werner and Dring 2011). However, they do present opportunities for the production of different products in biorefinery cascades. *P. palmata* is plentiful enough that it can be collected and screw-pressed at pilot scale. The major part of the original biomass metabolite suite remained in the solid residue, indicating that the juice fraction is of limited use for future processing and contains little DM. If metabolite utilization is not feasible or desirable, *P. palmata* can also be successfully ensiled without additives and used as a potential bioenergy feedstock. Once ensiled, though, a large proportion of the DM is lost in the relatively high volume of effluent (compared to terrestrial biomass) which may limit opportunities for its use in this fashion in the future. Further work is required to investigate chemical partitioning in the ensiled liquid and solid fractions and to establish if there are any additional compounds present in the effluent that may have biorefinery/therapeutic benefits.
